# Relocation of active site carboxylates in major facilitator superfamily multidrug transporter LmrP reveals plasticity in proton interactions

**DOI:** 10.1038/srep38052

**Published:** 2016-12-05

**Authors:** Asha V. Nair, Himansha Singh, Sagar Raturi, Arthur Neuberger, Zhen Tong, Ning Ding, Kelvin Agboh, Hendrik W. van Veen

**Affiliations:** 1 Department of Pharmacology, University of Cambridge, Tennis Court Road, Cambridge CB2 1PD, UK

## Abstract

The expression of polyspecific membrane transporters is one important mechanism by which cells can obtain resistance to structurally different antibiotics and cytotoxic agents. These transporters reduce intracellular drug concentrations to subtoxic levels by mediating drug efflux across the cell envelope. The major facilitator superfamily multidrug transporter LmrP from *Lactococcus lactis* catalyses drug efflux in a membrane potential and chemical proton gradient-dependent fashion. To enable the interaction with protons and cationic substrates, LmrP contains catalytic carboxyl residues on the surface of a large interior chamber that is formed by transmembrane helices. These residues co-localise together with polar and aromatic residues, and are predicted to be present in two clusters. To investigate the functional role of the catalytic carboxylates, we generated mutant proteins catalysing membrane potential-independent dye efflux by removing one of the carboxyl residues in Cluster 1. We then relocated this carboxyl residue to six positions on the surface of the interior chamber, and tested for restoration of wildtype energetics. The reinsertion at positions towards Cluster 2 reinstated the membrane potential dependence of dye efflux. Our data uncover a remarkable plasticity in proton interactions in LmrP, which is a consequence of the flexibility in the location of key residues that are responsible for proton/multidrug antiport.

The ability of microbes to develop resistance to cytotoxic drugs, and to adapt rapidly to changes in the exposure to these compounds is an extremely important medical problem^1^. Drug resistance can be specific for a single drug or class of drugs, or occur simultaneously for a wide variety of toxic compounds that are structurally and functionally unrelated. The latter phenomenon is called multidrug resistance, and is recognised as an important mechanism of bacterial resistance to valuable clinical antibiotics. In many cases, the development of multidrug resistance is due to the enhanced expression levels of drug pumps in the cell^2^. These pumps mediate the extrusion of antimicrobials across the cell envelope, away from intracellular targets. Enzyme-ligand interactions are usually based on very specific interactions that allow discrimination between enantiomers of the same ligand. The ability of multidrug transporters to recognise a broad spectrum of structurally unrelated drugs is intriguing.

Multidrug transporters have been reinvented on multiple occasions in the course of evolution, and are found in six different protein families: the ATP-binding cassette (ABC) superfamily, resistance-nodulation-cell division (RND) family, multiple antibiotics and toxin extrusion (MATE) family, small multidrug resistance (SMR) family, proteobacterial antimicrobial compound efflux (PACE) family, and major facilitator superfamily (MFS)^3^,^4^. It is generally accepted that members of the ABC family couple drug efflux to ATP binding and hydrolysis, whereas the members of the other 5 families operate by a secondary-active transport mechanism in which efflux is coupled to electrochemical ion gradients that exist across the plasma membrane. However, detailed studies on the energetics of gram-positive *Lactococcus lactis* LmrA and gram-negative *Escherichia coli* MsbA have shown that, in addition to the nucleotide dependence, the efflux reactions catalysed by these ABC exporters are also coupled to electrochemical ion gradients^5^,^6^. This analogy in energy coupling between classes of multidrug transporters makes studies on the underlying mechanisms of ion coupling highly relevant.

LmrP is an MFS multidrug transporter from *L. lactis* that is relatively well characterised at the functional level^7^. The protein contains 408 amino acids in 12 transmembrane helices (TMs) and connecting loops which, similar to many other MFS members, are organised in 2 bundles of 6 TMs in an N- and C-terminal membrane domain^8^. LmrP utilises both the chemical proton gradient (ΔpH, interior alkaline) and membrane potential (Δψ, interior negative) components of the proton motive force (Δp)^9^ to drive electrogenic (membrane potential-dependent) propidium efflux from the cell via apparent propidium^2+^/3H^+^ antiport^10^. In an inward-facing three-dimensional homology model of LmrP^11^ based on the crystal structure of the glycerol-phosphate/phosphate antiporter GlpT^12^, the N- and C-terminal membrane domains are suggested to form a large interior chamber containing three carboxyl residues (Asp-142, Asp-235 and Glu-327) that are surrounded by polar and aromatic residues. These carboxyl residues are predicted to be organised into two catalytic clusters, one containing Asp-235 and Glu-327 in the C-terminal domain (Cluster 1) with a distance of ~4.7 Å between the side chain centres and with a known ability to coordinate Ca^2+ ^13, and the other containing Asp-142 in the N-terminal domain (Cluster 2) at a distance of ~16.7 Å from Asp-235 and ~16.1 Å from Glu-327 (Fig. 1A). The observed stoichiometry for the electrogenic antiport of divalent cationic propidium (3H^+^/propidium^2+^) in LmrP, and the effect of carboxyl residue replacements on this stoichiometry suggest that in the propidium transport reaction, Asp-235 and Glu-327 (Cluster 1) are important for the interaction of LmrP with 2 protons and the substrate, whereas Asp-142 (Cluster 2) is dedicated to the interaction with 1 proton only^10^. Although each of the two clusters interacts with 1 proton in the ethidium transport reaction, none of the three catalytic carboxylates is essential for the interaction of LmrP with this monovalent cation^10^.

To further investigate the functional properties of catalytic carboxylates in LmrP, we changed the composition of Cluster 1 by removal of Glu-327, yielding mutant proteins with altered transport properties, and reinserted this carboxylate at 6 other positions in the interior chamber (Fig. 1B). Among the tested positions, the relocation to positions Thr-331 and Ala-355 towards Cluster 2, restored functional properties of wild-type (WT) LmrP. Our data highlight a flexibility in the location of catalytic carboxylates in this multidrug transporter.

## Results

### Insertion of carboxyl residues in E327Q LmrP

In transport experiments in the drug-hypersensitive *Lactococcus lactis* NZ9000 *∆lmrA ∆lmrCD* expressing LmrP proteins, the ionophores nigericin and valinomycin were employed to selectively manipulate the composition and magnitude of the *∆*p. Nigericin mediates the antiport of H^+^ and K^+^ down their concentration gradients, thereby selectively dissipating *∆*pH in an electroneutral (membrane potential-independent) manner. Furthermore, valinomycin mediates electrogenic uniport of K^+^, allowing the electrophoretic uptake of K^+^ in cells with dissipation of the *∆*ψ^14^. ATP-depleted cells were first pre-equilibrated with 2 μM propidium or ethidium, after which the efflux reaction was initiated with the addition of 25 mM glucose in the absence and presence of valinomycin and/or nigericin. The control experiments in Fig. 2A and B show that WT LmrP mediates *∆*ψ and *∆*pH-dependent substrate/proton antiport for both propidium and ethidium. As the insertion of extra carboxyl residues with additional proton coupling would yield LmrP mutants whose transport reaction is dependent on *∆*ψ and *∆*pH as seen for WT LmrP, such mutants might not be readily detectable. Therefore, we performed our insertion studies in the His_6_-tagged LmrP mutant in which the catalytic carboxyl residue Glu-327 is replaced by the corresponding neutral amide (E327Q). This mutant was found to mediate electroneutral proton-substrate antiport for propidium, but electrogenic transport for ethidium (Fig. 2C,D). These data have been obtained before^10^ and are in line with the current results.

**Figure 1:**
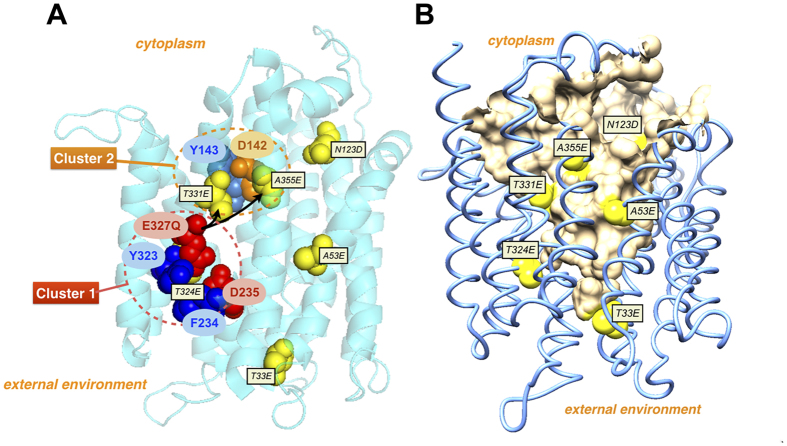
Structure models of inward-facing LmrP. (**A**) LmrP contains two catalytic clusters, Cluster 1 containing Asp-235 and Glu-327 in the C-terminal domain (red dotted circle), and Cluster 2 containing Asp-142 in the N-terminal domain (orange dotted circle)^10^,^11^. In addition to these carboxyl residues (in red or orange), the clusters also contain one or more aromatic residues (in blue) and polar residues (not shown). To investigate the functional role of these catalytic carboxylates and clusters, we changed the composition of Cluster 1 by removal of Glu-327 and relocation of this carboxyl residue to 6 positions (in yellow) in the interior chamber. Positions for reinsertion of Glu-327 are indicated in smaller, yellow-filled, black rectangles. One of these reinsertions, T324E, is partially hidden behind Y323 and E327Q in the figure; the N-terminus is also hidden. Helices in front of both clusters (TM 7 [Gln-213 - Ile-231] and TM 12 [Lys-376 - Asn-406] containing the C-terminus) are not shown for clarity of presentation. Relocations of Glu-327 from Cluster 1 to positions Thr-331 and Ala-355 near Cluster 2 yielded LmrP mutants with a similar functionality as WT LmrP. These relocations are indicated by black arrows. (**B**) Reinserted carboxylates (in yellow) are located on the surface (in orange) of the interior chamber in a full model of LmrP. Molecular graphics and analyses were performed with UCSF Chimera package (v 1.10.2) and PyMOL Molecular Graphics System (v.1.74). The interior chamber was calculated using CASTp cavity analysis^29^.

An additional carboxyl residue was then introduced in various locations on the surface of the internal binding cavity in E327Q LmrP. Locations Thr-33, Ala-53, Asn-123, Thr-324, Thr-331 and Ala-355 were selected using the inward-facing three-dimensional LmrP homology model that was previously constructed based on the crystal structure of the glycerol-3P/Pi antiporter GlpT^11^ (Fig. 1A,B). Positions were chosen with respect to their proximity to the catalytic carboxylates Asp-235 (located in Cluster 1) and Asp-142 (located in Cluster 2). In our structure model, (i) position 324 is located in closer proximity to Cluster I [Glu-327 (~4.2 Å)/Asp-235 (~6.1 Å)] than Cluster II [Asp-142 (~16.6 Å)], (ii) position 331 is located approximately halfway between Cluster I [Glu-327 (~9.4 Å)/Asp-235 (~11.3 Å)] and Cluster II [Asp-142 (~9.7 Å)], and (iii) position 355 is located in closer proximity to Cluster II [Asp-142 (~11.2 Å)] than Cluster I [Glu-327 (~15.9 Å)/Asp-235 (~17.4 Å)]. Positions 33, 53 and 123 are further away from the two clusters, and are located at the opposite side of the interior chamber (Fig. 1B). Secondary mutations T33E, A53E, N123D, T324E, T331E and A355E were subsequently generated in the background of E327Q LmrP. Western blot analysis of total membrane proteins from inside-out membrane vesicles showed that except for A53E/E327Q LmrP, which was expressed at 43% of the expression level of WT LmrP, the other mutants showed equal amounts of accumulated protein in the plasma membrane compared to WT LmrP (Fig. 3A).

**Figure 2:**
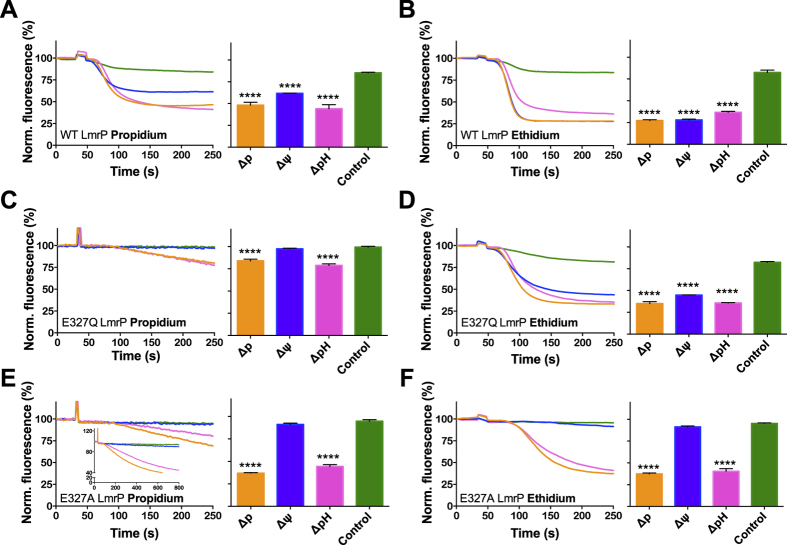
Energetics of dye efflux by WT LmrP, E327Q LmrP and E327A LmrP. ATP-depleted cells expressing WT LmrP (**A**,**B**) and the single mutants E327Q (**C**,**D**) or E327A (**E**,**F**) were diluted to OD_660_ of 0.5 in KPi buffer (pH 7.0) supplemented with 5 mM MgSO_4_ and pre-equilibrated with 2 μM propidium (**A**,**C**,**E**) or ethidium (**B**,**D**,**F**). Ionophores nigericin or valinomycin or both were added 3 min prior to the addition of glucose to measure transport in the presence of *∆*ψ or *∆*pH only, or without *∆*p (Control), respectively. The normalised fluorescence start level before glucose addition is set at 100% to facilitate direct comparison of data for LmrP mutants with different dye preloading activities. Histograms display the mean ± s.e.m. of dye fluorescence end levels in 3 independent experiments with independently prepared batches of cells compared to control for which the *∆*p was absent (one-way analysis of variance; ****P < 0.0001), and demonstrates the reproducibility of data obtained in these assays. *Inset* in (**E**) shows the same data as the main figure at a longer time scale.

**Figure 3:**
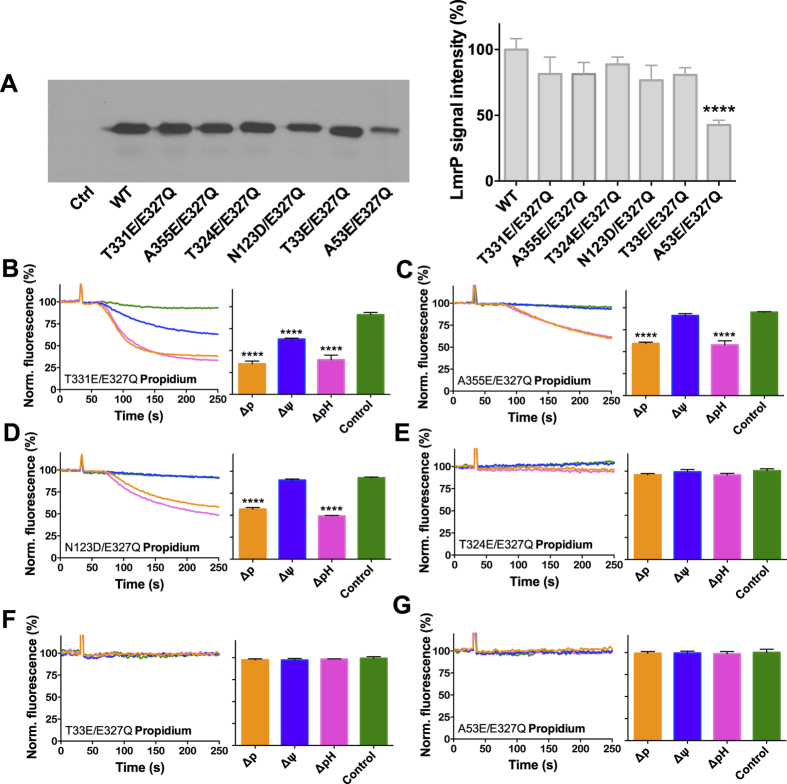
Expression and activity of E327Q LmrP mutants with a reinserted carboxyl residue. (**A**) Immunodetection of total membrane proteins in plasma membrane vesicles (10 μg of protein/lane) containing WT LmrP or mutants LmrP, or no LmrP protein (Ctrl). Proteins were transferred from SDS-PAGE gel to HybondP membrane by electroblotting and probed with anti-His-tag and secondary antibodies. For clarity of presentation, blot was cropped in Adobe Illustrator CC 2015.3; full-length blot is available at http://dx.doi.org/10.17863/CAM.6219. (**B**) Cells expressing the T331E/E327Q double mutant LmrP were diluted in KPi buffer (pH 7.0) supplemented with 5 mM MgSO_4_ and pre-equilibrated with 2 μM propidium. (**C–G**) Transport experiments as in (**B**) for A355E/E327Q (**C**), N123D/E327Q (**D**), T324E/E327Q (**E**), T33E/E327Q (**F**), and A53E/E327Q (**G**). See legend to Fig. 2 for further details. Data represent observations in 3 or more independent experiments with independently prepared batches of membrane vesicles or cells. Values in histograms are expressed as mean ± s.e.m. (one-way analysis of variance; ****P < 0.0001).

To examine the energetics of LmrP E327Q mutants with an inserted carboxyl residue, propidium efflux in intact cells was measured in the absence or presence of ionophores as described for Fig. 2. The introduction of E at position 331 substantially restored electrogenic propidium transport pointing to the ability of the T331E/E327Q double mutant to utilise both the ∆pH and the ∆ψ in the efflux reaction (Fig. 3B). In contrast, the double mutants A355E/E327Q and N123D/E327Q showed propidium efflux activity in the presence of either the ∆p or the ∆pH, but negligible transport activity in the presence of the ∆ψ only (Fig. 3C,D). These mutants therefore display the same phenotype as the single mutant E327Q background in which the mutations were generated. The double mutants T324E/E327Q, T33E/E327Q and A53E/E327Q (Fig. 3E–G) did not mediate detectable propidium efflux in the presence of ∆p or its components. As all mutants were expressed in the plasma membrane (Fig. 3A), these data suggest that an acidic residue in position 33, 53 and 324 somehow interferes with the propidium transport reaction.

### Insertion of carboxyl residues in E327A LmrP

To examine changes in ethidium transport for the carboxylate insertion mutants, Glu-327 was replaced by A in His_6_-tagged LmrP. In control experiments, the single mutant E327A showed active propidium and ethidium efflux in the presence of the *∆*p (interior negative and alkaline) or its *∆*pH component, while transport activity was reduced to a negligible level with the *∆*ψ only (Fig. 2E,F). Thus, E327A LmrP mediates electroneutral transport of these cationic dyes, and provides an ideal background to test for carboxylate insertions that restore the *∆*ψ dependence in ethidium transport.

**Figure 4:**
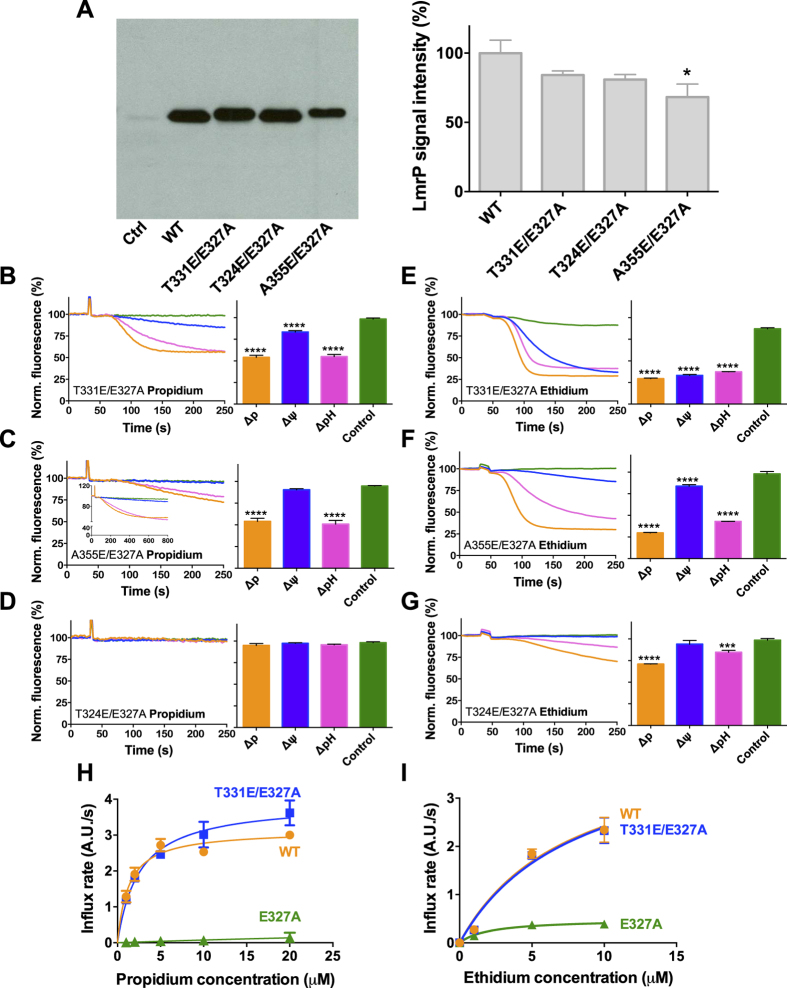
Expression and activity of E327A LmrP mutants with a reinserted carboxyl residue. (**A**) Immunodetection of total membrane proteins in plasma membrane vesicles (10 μg of protein/lane) containing WT LmrP or mutants LmrP, or without LmrP protein (Ctrl). Full-length blot is available at http://dx.doi.org/10.17863/CAM.6219. (**B–G**) Cells expressing the double mutants T331E/E327A (**B**,**E**), A355E/E327A (**C**,**F**) and T324E/E327A (**D**,**G**) were diluted in KPi buffer (pH 7.0) supplemented with 5 mM MgSO_4_, and were pre-equilibrated with 2 μM propidium (**B**–**D**) or ethidium (**E**–**G**). See legend to Fig. 2 for further details (*P ≤ 0.05). *Inset* in (**C**) shows the same data as the main figure at a longer time scale. (**H**,**I**) Kinetics of facilitated propidium and ethidium influx by WT LmrP and LmrP mutants. ATP-depleted cells expressing WT LmrP (●), the single mutant E327A (▲) and double mutant T331E/E327A (■) were diluted in KPi buffer (pH 7.0) supplemented with 5 mM MgSO_4_, after which propidium (**H**) or ethidium (**I**) was added. The rates of influx were calculated over the initial 30 s and plotted against the dye concentration as the mean ± s.e.m; values in histograms are expressed as mean ± s.e.m. (one-way analysis of variance; *P < 0.05; ***P < 0.001; ****P < 0.0001). The error bars for some of the data points in (**H**,**I**) are too small to be displayed, and are hidden behind the data point symbols. Data represent observations in 3 or more independent experiments with independently prepared batches of membrane vesicles or cells.

The carboxylate substitutions at positions 324, 355 and 331 were selected for further experiments as they represent the three categories of mutants that were encountered in the propidium transport experiments in E327Q LmrP (Fig. 3): transport-inactive, tolerated but silent, and catalytically-active relocations of Glu-327, respectively. Double mutants T331E/E327A and A324E/E327A were equally well expressed as WT LmrP in the plasma membrane of *L. lactis*, whereas A355E/E327A was expressed at 68% of the WT level (Fig. 4A). To test whether the introduction of the carboxyl residues on the surface of the internal cavity can restore the ability of the E327A mutant to mediate ∆ψ-dependent ethidium transport, similar substrate efflux assays were carried out as described for the E327Q proteins (Fig. 3). In experiments with propidium, comparable results were obtained in the E327A background as for E327Q (Fig. 4B–D). For ethidium transport, the introduction of E at position 331 and 355 in E327A LmrP, each supported ∆ψ-dependent transport (Fig. 4E,F). Although A355E/E327A showed a weaker activity in ∆ψ-dependent ethidium transport compared to T331E/E327A, the activity of A355E/E327A was significant compared to E327A LmrP (Fig. 2F)(P < 0.01). Mutant T324E/E327A transported ethidium (Fig. 4G) in a comparable fashion as E327A LmrP but at lower rates (Fig. 4B) suggesting that T324E is not catalytically active in ethidium transport in the E327A background but that the replacement is tolerated. Taken together, T331E and A355E are the only carboxyl residues in our test panel that impose electrogenicity on transport, for T331E, of propidium and ethidium transport, and for A355E, of ethidium transport only. These carboxylates are therefore most likely functional in proton coupling with these substrates.

The effect of the T331E replacement on the affinity of E327A LmrP for propidium and ethidium was evaluated by measuring the kinetics of facilitated substrate influx in ATP-depleted cells. Following the addition of propidium or ethidium at concentrations ranging from 1 μM to 30 μM, the rates of substrate uptake over the initial 30 s were determined. These rates were corrected for dye influx in control cells without LmrP expression, and were subsequently plotted against the substrate concentration (Fig. 4H,I). The propidium binding affinity of T331E/E327A LmrP (expressed as the substrate concentration K_t_ giving a half-maximal rate of transport V_max_) was similar in comparison to WT LmrP (K_t_ = 2.5 ± 0.4 μM versus 1.3 ± 0.2 μM). Similarly, no significant difference was observed for ethidium in this comparison (K_t_ = 8.1 ± 5.3 μM versus 7.7 ± 5.1 μM). Furthermore, the V_max_ values for dye uptake by T331E/E327A LmrP were similar to those obtained for WT LmrP (propidium, V_max_ = 3.9 ± 0.2 A.U./s versus 3.1 ± 0.1 A.U./s; ethidium, V_max_ = 4.3 ± 1.5 A.U./s versus 4.3 ± 1.4 A.U./s). As the rates of facilitated dye uptake by E327A LmrP were too low for kinetic analysis, the data suggest that insertion of E at position 331 in the E327A background restores the transport capabilities of the single mutant to those of WT LmrP, and hence, that T331E compensates for the loss of E at position 327.

### T331E substitution in WT LmrP

**Figure 5:**
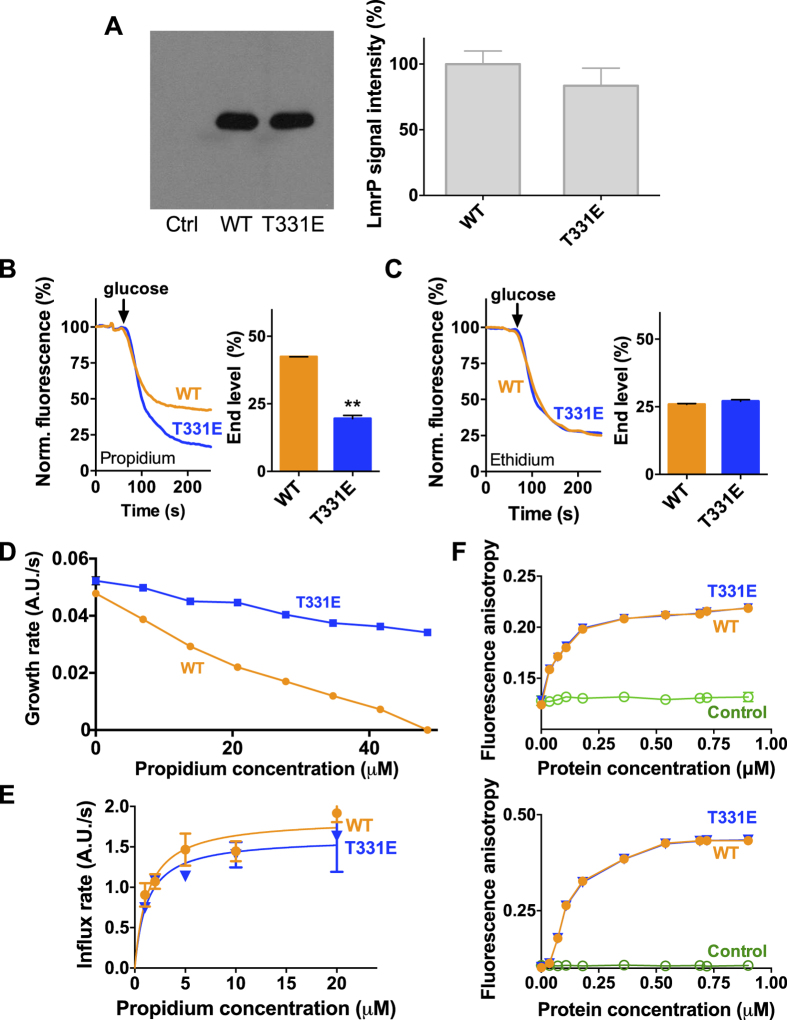
Comparison of the expression and activity of T331E LmrP versus WT LmrP. (**A**) Immunodetection of total membrane proteins in plasma membrane vesicles (10 μg of protein/lane) containing WT LmrP or T331E LmrP. Full-length blot is available at http://dx.doi.org/10.17863/CAM.6219. (**B**,**C**) Dye transport in intact cells. ATP-depleted cells expressing WT LmrP and the single mutant T331E were diluted in buffer, and pre-equilibrated with 2 μM propidium (**B**) or ethidium (**C**). Histograms show significant differences between fluorescence end levels for transport of propidium but not ethidium. (**D**) Measurement of the growth rate (A.U./s) of cell cultures as a function of propidium concentration shows that T331E LmrP expression confers increased resistance on the cells compared to WT LmrP. (**E**) Kinetics of facilitated propidium influx in ATP-depleted cells containing WT LmrP (●) or T331E LmrP (▼). (**F**) Propidium binding to WT LmrP and T331E LmrP. Purified protein in detergent solution was added stepwise to buffer containing 0.2 μM propidium (*top*) or 1 μM propidium (*bottom*), after which the fluorescence anisotropy was measured. Elution buffer without protein served as the control (○). Data represent observations in 3 or more independent experiments with independently prepared batches of membrane vesicles or cells. Values in data points and histograms are expressed as mean ± s.e.m. (**A**–**C**) unpaired student t-test; (**D**–**F**), one-way analysis of variance; **P < 0.01). The error bars for the data points in (**D**,**F**) are too small to be displayed, and are hidden behind the data point symbols.

As a carboxyl residue at position 331 is functionally active in drug/proton antiport in E327Q and E327A mutants, the effect of this replacement was also tested in the WT background (Fig. 5). The T331E mutant was generated in His_10_-tagged LmrP, and expressed to a similar level as WT (Fig. 5A). It was found that T331E LmrP mediates propidium efflux to a lower steady-state level compared to WT LmrP (Fig. 5B), which is consistent with the increased propidium resistance of T331E LmrP-expressing cells compared to WT LmrP-expressing cells (Fig. 5D). For ethidium, T331E LmrP showed a similar transport as WT LmrP (Fig. 5C). To delineate whether the enhanced propidium transport by T331E LmrP was due to enhanced proton coupling and/or enhanced interaction with propidium, the binding affinity of T331E LmrP for propidium was first measured using the facilitated influx assay in lactococcal cells as described for T331E/E327A LmrP (Fig. 4H). The K_t_ of T331E LmrP and WT LmrP for propidium was found not to be significantly different, 1.2 ± 0.4 μM and 1.3 ± 0.3 μM, respectively. Furthermore, propidium binding to purified T331E LmrP and WT LmrP was measured by fluorescence anisotropy (Fig. 5F). In this assay, propidium at a fixed concentration of 1 μM or 0.2 μM was titrated with increasing concentrations of purified protein in detergent solution until a steady level of fluorescence anisotropy was achieved. These fixed ligand concentrations are in the same range or 5 to 10-fold below the apparent K_t_ of Wt LmrP and T331E LmrP for propidium, and hence, enable propidium binding in a protein concentration-dependent, hyperbolic fashion (Fig. 5F). As the measurements yielded binding data for Wt LmrP and T331E LmrP that were overlapping, these data indicate that there was no significant difference in the propidium binding affinity of both proteins.

**Figure 6:**
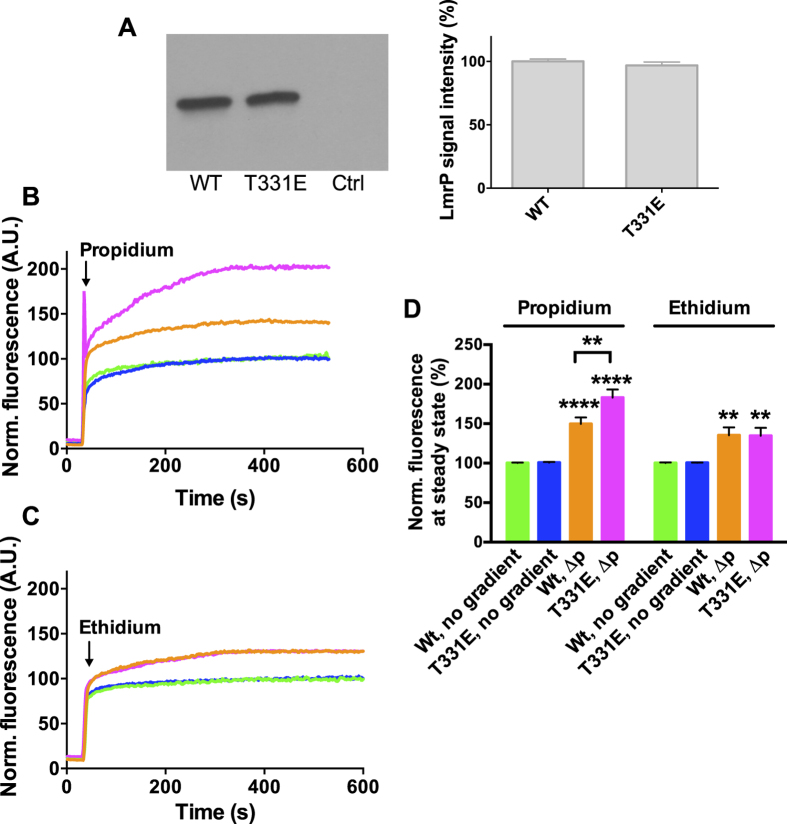
Dye transport in proteoliposomes. (**A**) Immunodetection of his-tagged protein shows that equal amounts of WT LmrP or T331E LmrP protein were incorporated in the proteoliposomal membrane, and that the empty control liposomes (Ctrl) were devoid of LmrP. Full-length blot is available at http://dx.doi.org/10.17863/CAM.6219. (**B–D**) Proteoliposomes were diluted to impose an artificial ∆p (interior positive and acidic) or no gradient. At the arrow, 2 μM propidium (**B**) or ethidium (**C**) was added, after which dye accumulation was measured over time by fluorimetry. Steady-state fluorescence levels (**D**) at approx. 400 s suggests a significantly elevated level of propidium accumulation for T331E LmrP compared to WT LmrP. Line colours in (**B**,**C**) are the same as bar colours in (**D**) and refer to the same experimental conditions. No significant difference was observed for ethidium in this comparison. Data represent observations in 3 or more independent experiments with independently prepared batches of proteoliposomes. The mean fluorescence level in the absence of *∆*p was set at 100%. Values in histograms are expressed as mean ± s.e.m. (**A**) unpaired student t-test; (**D)**, two-way analysis of variance; **P < 0.01; ****P < 0.0001).

The effect of the insertion of T331E in WT LmrP on the energetics of transport was tested in proteoliposomes in which purified LmrP or T331E LmrP were functionally reconstituted at equal levels in the phospholipid bilayer (Fig. 6A). In this system, dye accumulation was monitored by fluorimetry through inclusion of 1 mg/ml sheared calf thymus DNA in the lumen of the liposomes^6^. For the imposition of the ∆p, a transmembrane ∆pH (interior acidic) and ∆ψ (interior positive) were imposed artificially by the preparation of the (proteo) liposomes in NH_4_SCN containing buffer (pH 6.8), and subsequent 100-fold dilution in NH_4_SCN-free buffer (pH 7.5), thus allowing the passive diffusion of NH_3_ and SCN^−^ to the external buffer^6^. With the addition of 2 μM propidium to the external buffer, a significantly higher ∆p-dependent accumulation of propidium was obtained in proteoliposomes containing T331E LmrP compared to WT LmrP, whereas the no gradient controls showed a similar low background-level fluorescence (Fig. 6B). In contrast, when 2 μM ethidium was added in the assay a similar level of ethidium accumulation was observed for both T331E and WT LmrP compared to the no gradient controls (Fig. 6C). The basic thermodynamic relationship between the driving force and the propidium concentration gradient across the proteoliposomal membrane at equilibrium is given by:


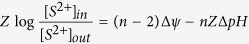


in which *Z* = 2.3RT/F = approx. 59 mV at 25 °C, and *n* is the number of H^+^ that participates in the antiport reaction. The elevated propidium accumulation level in proteoliposomes for ∆p-dependent transport by T331E LmrP compared to WT LmrP (Fig. 6D) therefore indicates a higher value of *n* for T331E LmrP in the propidium transport reaction. This conclusion is also in agreement with propidium efflux measurements in cells showing efflux to lower end levels for T331E LmrP compared to WT LmrP (Fig. 5B).

Taken together, these data suggest that the enhanced propidium transport activity of T331E LmrP compared to WT LmrP is not due to enhanced interaction of the mutant with propidium but that the extra carboxyl residue introduced at position 331 functions as an additional proton-binding site in the propidium transport reaction.

## Discussion

Although previous work identified three catalytic carboxylates in LmrP (Asp-142, Asp-235 and Glu-327), it was suggested that these carboxylates only form two proton binding groups in the transport reaction for monovalent ethidium^10^. In the presence of ethidium, Glu-327 and Asp-235 in wild-type LmrP are proposed to form a carboxyl-carboxylate pair containing Asp-235 as a single proton acceptor moiety that is stabilized through hydrogen bonding with the adjacent undissociated carboxyl moiety in Glu-327 or amide moiety in E327Q. This stabilization is likely to elevate the pKa of Asp-235 to a value closer to the physiological intracellular pH where Asp-235 becomes a functional proton binding group. Therefore, the Δp dependence of ethidium transport is similar for wildtype (Glu-327) and the E327Q mutant in the experiments in Fig. 2B and D, and reflects apparent electrogenic 2H^+^/ethidium^+^ antiport. However, when E327Q is replaced by E327A the lower pKa of Asp-235 would disable this carboxyl group in proton binding under physiological conditions, yielding apparent electroneutral 1H^+^/ethidium^+^ antiport as observed in our data (Fig. 2F). When divalent cationic propidium is bound on the surface of the interior chamber of LmrP, Glu-327 and Asp-235 might not directly interact with each other due to changes in the distance and geometry of their side chains compared to the ethidium-bound state, and both carboxyl residues now function as independent proton acceptor moieties^10^. Such changes in side-chain interactions in response to the binding of different substrates have been observed in co-crystal structures of the multidrug binding transcriptional repressor QacR^15^,^16^. Together with Asp-142, the independent activities of Asp-235 and Glu-327 in LmrP now result in apparent electrogenic 3H^+^/propidium^2+^ antiport. Consistent with the experimental data for E327Q (Fig. 2C) and E327A (Fig. 2E), this stoichiometry is reduced to apparent electroneutral 2H^+^/propidium^2+^ antiport upon the loss of Glu-327.

Using rational design, we modified the relative distance between the membrane-embedded carboxyl residues in the interior chamber of LmrP. Out of the 6 tested positions, T331E recovered WT-like electrogenic transport for propidium and ethidium in E327A LmrP, whereas A355E recovered electrogenic transport for ethidium but not for propidium in this background. The T331E substitution also recovered WT-like propidium transport capabilities in the E327Q background. These findings point to a functional redundancy between T331E and Glu-327. In these experiments, the deleted Glu-327 carboxylate migrates from being close to Asp-235 (~4.7 Å) to positions where it has a large distance from Asp-235, ~11.3 Å in the case of T331E, and ~17.4 Å in the case of A355E, and where this carboxylate is functionally active in proton coupling. This result demonstrates the plasticity in the location of proton-binding moieties in LmrP. As the T331E and A355E mutations involve the relocation of Glu-327 from Cluster 1 towards Cluster 2, our data point to the existence of an area in the interior chamber, with Cluster 1 and 2 at its boundaries, in which carboxylates are active in proton-coupling.

The transport measurements indicate that the insertion of T331E in WT LmrP allows the mutant transporter to sustain a larger inwardly directed propidium concentration gradient in cells, and outwardly directed propidium concentration gradient in proteoliposomes in which the transporter is inserted in an inside-out fashion. Together with the observations on ∆p-dependent propidium transport in proteoliposomes, these results strongly suggest that T331E acts as proton binding group in addition to the existing Asp-142, Asp-235 and Glu-327. The established stoichiometry of 3H^+^/propidium^2+^ for WT LmrP^10^ will therefore most likely increase to 4H^+^/propidium^2+^. This conclusion is consistent with recent observations on the MFS transporter MdfA from *E. coli* showing that an increase in proton/drug stoichiometry requires an additional acidic residue in the multidrug recognition pocket of this transporter^17^. As the insertion of T331E in Wt LmrP did not enhance the ethidium concentration gradients in cells and proteoliposomes, T331E might share a functional similarity with Glu-327 in that neither of these carboxylates appears to play an active role in proton coupling during the ethidium transport reactions by T331E LmrP and Wt LmrP (see discussion for Glu-327 in Wt LmrP above). Our detailed understanding of these phenomena will require the high-resolution structural elucidation of the ethidium-LmrP and propidium-LmrP complexes in which the side-chain interactions of Asp-142, Asp-235, Glu-327 and T331E in inward-facing and outward-facing states of LmrP are resolved.

Similar to the native catalytic carboxylates Glu-327 and Asp-235, the newly introduced T331E and A355E are located in the C-terminal half of LmrP. As none of the other substitutions at the positions 33, 53 and 123 in the N-terminal half was catalytically active, the data suggest that although, like MdfA, LmrP is promiscuous regarding the positions where carboxyl residues can be functionally inserted, a bias might exist towards positions in the C-terminal half. This finding is reminiscent of previous observations on the MFS oxalate transporter OxlT, a GlpT homolog from *Oxalobacter formigenes*^18^. The ligand-binding residues in OxlT all lie in the C-terminal domain (on TM8 and TM11), whereas those in the glycerol-phosphate/phosphate antiporter GlpT lie at symmetrical positions (on TM1 and TM7) at the interface between the N- and C-terminal halves^19^.

Secondary-active multidrug transporters can confer drug resistance on the cell by facilitating active drug export across a plasma membrane that provides a barrier for passive influx of drugs^20^. Drugs can be expelled against their concentration gradient or lipid-water partition coefficient by coupling drug extrusion to the movement of protons and/or sodium ions down their electrochemical gradient. Our understanding of the mechanistic principles behind this biochemical coupling process requires knowledge of the ion translocation pathway(s), as well as the link between ion movement and conformational changes within the transporter that result in alternating access of drug binding surfaces. The mechanism of LacY has been intensely studied^21^,^22^,^23^, and was found to rely on proton binding to critical carboxylates, the dissociation of which is based on competitive binding of the cationic side chain of a neighbouring basic residue. The location of these catalytic carboxylates is therefore very precise and crucial for transport activity. A similar type of mechanism is also relevant for proton coupling in the trimeric RND multidrug transporter AcrB, where Lys-940, Arg-971, Asp-407 and Asp-408 are part of a proton relay network within the membrane domain of each AcrB protomer. This network is functionally coupled to drug binding at, and drug translocation through, the periplasmic domains in the AcrB trimer^24^. Previous studies on basic residues in LmrP highlighted the important role of Arg-260 and Lys-337 in the functional properties of this transporter^25^. Although these residues are predicted in our structure model to be oriented towards the head group region of the outer leaflet (Arg-260) and inner leaflet (Lys-357) of the cytoplasmic membrane, helical rotations during the transport cycle or a small 1 to 2 residue shift of the polypeptide in our structure model could place these basic residues in the central cavity where they might directly interact with the catalytic carboxylates. However, the protonation state of the catalytic carboxylates in LmrP could also be regulated by mechanisms that do not require the proximity of basic residues, e.g. by conformation-dependent changes in the physico-chemical properties of the local environment near the catalytic clusters. If relevant, the lack of AcrB and LacY-type stereospecific proton dissociation mechanisms in LmrP could be one of the reasons why this transporter tolerates relocations of carboxylates between cluster regions in which these carboxylates are catalytically active in proton/drug antiport.

In conclusion, our data suggest that LmrP exhibits a plasticity in proton interactions due to the intrinsic flexibility in the location of the key residues that are responsible for proton/drug exchange. This flexibility enables LmrP to rapidly evolve its polyspecificity through the acquisition of mutations while retaining proton/drug antiport activity.

## Methods

### Bacterial strains, protein expression, and site-directed mutagenesis

The His_6_-tagged *lmrP* wild type and mutant genes were cloned in the pNZ8084 plasmid downstream of the *nisA* promoter region^7^,^26^. Plasmids were maintained in the drug-hypersensitive *L. lactis* NZ9000 Δ*lmrA* Δ*lmrCD*^27^ lacking endogenous multidrug efflux pumps, which was grown at 30 °C in M17 broth (Oxoid) supplemented with 25 mM glucose and 5 μg/ml chloramphenicol. The cells were grown to an OD_660_ of 0.5–0.6 after which the protein expression was induced for 2 h by the addition of a 1:1000 dilution of nisin A-containing supernatant of the nisin-producing *L. lactis* strain NZ9700^26^ (approx. final concentration of 10 pg/ml of nisin A). The construction of *lmrP* mutants was carried out using the QuikChange method (Agilent Technologies) with mutagenic primer pairs as listed in Table 1. DNA was sequenced to ensure that only the intended changes were introduced.

### Transport measurements in intact cells

Cells were harvested by centrifugation at 6,500 x g for 10 min at 4 °C and washed once with 50 mM KPi (pH 7.0) containing 5 mM MgSO_4_. Harvested cells were then de-energised in the presence of 0.5 mM of the uncoupler dinitrophenol for 30 min at 30 °C to deplete ATP^28^. The de-energised cells were subsequently washed 3 times with ice-cold buffer, resuspended to an OD_660_ of 5.0, and kept on ice to maintain their viability. For monitoring active efflux, cells were diluted in prewarmed buffer to OD_660_ of 0.5, and equilibrated with 2 μM of either ethidium or propidium (fluorescence level of ~150 A.U. was obtained for propidium and up to ~250 A.U. for ethidium). Once the saturation level was obtained, 25 mM glucose was added as the source of energy and active efflux of the dye was measured by fluorimetry. The data were normalised by taking the fluorescence start level before the addition of glucose as 100% for direct comparison of data for LmrP mutants with different dye preloading activity. Where indicated, valinomycin was added 3 min before glucose addition at a concentration of 0.5 μM. Nigericin was added at a concentration of 0.5 μM for E327Q mutants and 1 μM for E327A mutants. Fluorescence measurements were performed in a Perkin Elmer LS-55B at λ_excitation_ of 500 nm and λ_emission_ of 580 nm with slit widths of 5 and 10 nm, respectively, for ethidium, and λ_excitation_ at 535 nm and λ_emission_ at 617 nm with slit widths of 10 nm each for propidium. For measurements of the kinetics of facilitated dye influx, cells were diluted 10-fold in 2 ml of buffer. Propidium or ethidium was added to final concentrations between 1 μM and 10 μM, and influx was monitored over the first 100 s.

### Preparation of inside-out membrane vesicles and protein purification

Inside-out membrane vesicles were prepared using as Basic Z 0.75-kW Benchtop Cell Disruptor as described^10^. The expression levels of the his-tagged LmrP proteins in the lactococcal plasma membrane vesicles were compared on immunoblot that was probed with primary mouse anti-polyhistidine tag antibody (Sigma-Aldrich, Cat. No.: H1029) and secondary goat anti-mouse antibody (Sigma-Aldrich, Cat. No.: A4416). LmrP signals were quantified by densitometric analysis of three independent blots using ImageJ software, version 1.43 (National Institutes of Health). For clarity of presentation, immunoblots in main figure panels were cropped in Adobe Illustrator CC 2015.3. The The complete immunoblots are available at http://dx.doi.org/10.17863/CAM.6219.

Membrane vesicles were solubilised at a protein concentration of 5 mg/ml in a solubilisation buffer containing 50 mM KPi (pH 8.0), 100 mM NaCl, 10% (v/v) glycerol, 1 mg/ml protease inhibitor cocktail and 1% *β*-D-dodecyl maltoside (DDM) (Melford and Anatrace). The solubilisation sample was incubated on a rotating wheel for 4 h at 4 °C. Insoluble material was removed by ultracentrifugation at 125,000 x g for 1 h at 4 °C. The supernatant was added to nickel-nitrilotriacetic acid (Ni-NTA, 1 ml of resin per 10 mg of his-tagged protein)-agarose resin (Sigma), previously equilibrated by two washes with five resin volumes of Milli-Q water and two washes with five resin volumes of Buffer A (50 mM KPi (pH 8.0), 100 mM NaCl, 10% (v/v) glycerol, 0.05% (w/v) DDM and 20 mM imidazole). The resin-binding mix was incubated on a rotating wheel at 4 °C overnight. The resin was transferred to a 2 ml Bio-spin chromatography column (Bio-Rad), washed with two column volumes of Buffer A and two column volumes of Buffer B (50 mM KPi (pH 7.0), 100 mM NaCl, 10% (v/v) glycerol, 0.05% (w/v) DDM and 20 mM imidazole), and eluted with two resin volumes of Buffer C (Buffer B supplemented with 200 mM imidazole and 5% glycerol). Protein concentrations were determined using the Micro-BCA assay kit (Pierce).

### Transport measurements in proteoliposomes

Purified LmrP WT or LmrP T331E protein was functionally reconstituted into liposomes by described methods^6^ in preparation buffer (pH 6.8) containing 10 mM K-Hepes, 100 mM K_2_SO_4_, 100 mM NH_4_SCN, and 1 mg/ml of sonicated calf thymus DNA (Trevigen). Purified protein was added at a lipid:protein ratio of 50:1 (w/w) to Triton X-100-destabilised liposomes, and the mixture was incubated for 30 min at room temperature. Detergent was removed by incubation with SM2 biobeads as described^6^. Subsequently, the proteoliposomes were incubated for 15–20 min with DNase (10 μl/ml) in the presence of 10 mM MgSO_4_ to remove any DNA contamination from the lipid bilayer, collected by centrifugation at 165,145 × g for 30 min, resuspended in 150–200 μl of preparation buffer, and used immediately in transport assays. For measurements of ethidium and propidium transport in (proteo)liposomes in the presence of an imposed Δp (inside acidic and negative), the proteoliposomes were diluted 100-fold in dilution buffer (pH 7.5) containing 10 mM K-Hepes and 100 mM K_2_SO_4_ in a total reaction volume of 2 ml. Ethidium or propidium was added to a final concentration of 2 μM, and transport was monitored in a Perkin Elmer LS-55B fluorimeter.

### Fluorescence anisotropy

Protein purification was done as described under “Preparation of inside-out membrane vesicles and protein purification” and the purified protein was diluted to a concentration of 0.4 mg/ml in 2 ml of 50 mM KPi buffer (pH 7.0) supplemented with 100 mM NaCl, 0.05% DDM, and 1 μM or 0.2 μM propidium. For propidium binding measurements, the purified protein was added in 3 steps of 3.45 μg each, followed by 1 step of 7.0 μg, 3 steps of 17.3 μg, 1 step of 3 μg and 1 step of 17 μg. Equivalent amounts of elution buffer (Buffer C under “Preparation of inside-out membrane vesicles and protein purification”) were added in a protein-free control sample.

### Drug resistance

*L. lactis* was grown in M17 medium supplemented with 25 mM glucose and 5 μg/ml chloramphenicol. After 16 h of growth at 30 °C, cell cultures were used to inoculate fresh medium at a 1:50 (v/v) dilution. At OD_660_ of 0.5, protein production was initiated through the addition of 10 pg/ml nisin. Protein expression was induced for 2 h, after which the culture was diluted 1:10 (v/v) in fresh medium supplemented containing 10 pg/ml nisin in the wells of a 96-well plate. Propidium was then added at concentrations as indicated in Fig. 5D. The wells were covered with 25 μl of sterile mineral oil to reduce evaporation of water. The growth of cultures in the wells at 30 °C was recorded by measuring the OD_660_ in a Versamax microplate reader (Molecular Devices) over a time period of 16 h. Cultures were grown in triplicates for each propidium concentration. The slopes of the exponential phase in growth curves were determined from the linear increase in a log10(OD_660_) versus time plot, and were plotted against the corresponding propidium concentration in Fig. 5D.

### Statistical analyses

The data points plotted for immuno blots and fluorescence studies are obtained in at least 3 independent experiments using separate batches of intact cells, membrane vesicles, or proteoliposomes and statistical parameters are plotted using GraphPad Prism 6 software. Histograms display the mean ± s.e.m., and demonstrate the reproducibility of the experiments. Means of the fluorescence end levels of Δp, Δψ and ΔpH-dependent dye transport in cells were compared with that of the control. Statistical analyses were performed using one-way analysis of variance (ANOVA). For Figs 5A–C and 6A the two-tailed student t-test was used. In proteoliposomes (Fig. 6D), steady-state levels of the normalised fluorescence were compared with that of the control without the imposed Δp. These analyses were performed by two-way ANOVA holm-sidak’s multiple comparison test. In the histograms, significance is shown by asterisks: ****P < 0.0001; ***P < 0.001; **P < 0.01; *P < 0.05; no asterisk, not significant. For studies on energetics of transport, asterisks directly above bars in the histograms refer to comparison with condition without Δp; asterisks above lines refer to specific comparisons.

### Data Availability

A previous version of this manuscript was withdrawn by the authors from publication in the Journal of Biological Chemistry (jbc.M115.694901). The figures in the current publication are based on new and original data which were generated in triplicate or more, and which have been deposited in the University of Cambridge data repository with accession link http://dx.doi.org/10.17863/CAM.6219.

## Additional Information

**How to cite this article**: Nair, A. V. *et al*. Relocation of active site carboxylates in major facilitator superfamily multidrug transporter LmrP reveals plasticity in proton interactions. *Sci. Rep.* **6**, 38052; doi: 10.1038/srep38052 (2016).

**Publisher's note:** Springer Nature remains neutral with regard to jurisdictional claims in published maps and institutional affiliations.

## Figures and Tables

**Table 1:** Sequences of PCR primers used for the generation of LmrP mutants.

T33E	Fw^a^: GGC ACC GTT TTT TCT TCA ATG **GAG** ATT TAT TAT AAT C Rv: G ATT ATA ATA AAT **CTC** CAT TGA AGA AAA AAC GGT GCC
A53E	Fw: C TTA TCT **GAG** GTG GCA ACT TTT GTC GCC Rv: GGC GAC AAA AGT TGC CAC **CTC** AGA TAA G
N123D	Fw: GTG ATA ACT GCT GGG **GAT** GCC ATG Rv: CAT GGC **ATC** CCC AGC AGT TAT CAC
T324E	Fw: GCA GGT ATT GTT TAT **GAG** TTG GGT CAG ATT GTT TAT ACC Rv: GGT ATA AAC AAT CTG ACC CAA **CTC** ATA AAC AAT ACC TGC
T331E	Fw: G GGT GAG ATT GTT TAT **GAG** CCA AGT GTT C Rv: G AAC ACT TGG **CTC** ATA AAC AAT CTC ACC C
A355E	Fw: GC GTT GCA **GAG** ATT AAA ATG CC Rv: GG CAT TTT AAT **CTC** TGC AAC GC

^a^Fw = forward primer, Rv = reverse primer, base substitution in codon in bold.
